# Genomic diversity, lifestyles and evolutionary origins of DPANN archaea

**DOI:** 10.1093/femsle/fnz008

**Published:** 2019-01-09

**Authors:** Nina Dombrowski, Jun-Hoe Lee, Tom A Williams, Pierre Offre, Anja Spang

**Affiliations:** 1 NIOZ, Royal Netherlands Institute for Sea Research, Department of Marine Microbiology and Biogeochemistry, and Utrecht University, P.O. Box 59, NL-1790 AB Den Burg, The Netherlands; 2 Department of Marine Science, University of Texas at Austin, Marine Science Institute, 750 Channel View Drive, Port Aransas, TX 78373, USA; 3 Department of Cell and Molecular Biology, Science for Life Laboratory, Uppsala University, P.O. Box 596, Husargatan 3, SE-75123 Uppsala, Sweden; 4 School of Biological Sciences, University of Bristol, Life Sciences Building, 24 Tyndall Avenue, Bristol, Bristol BS8 1TQ, UK

**Keywords:** DPANN, archaea, evolution, symbiosis, genomics, metabolism

## Abstract

Archaea—a primary domain of life besides Bacteria—have for a long time been regarded as peculiar organisms that play marginal roles in biogeochemical cycles. However, this picture changed with the discovery of a large diversity of archaea in non-extreme environments enabled by the use of cultivation-independent methods. These approaches have allowed the reconstruction of genomes of uncultivated microorganisms and revealed that archaea are diverse and broadly distributed in the biosphere and seemingly include a large diversity of putative symbiotic organisms, most of which belong to the tentative archaeal superphylum referred to as DPANN. This archaeal group encompasses at least 10 different lineages and includes organisms with extremely small cell and genome sizes and limited metabolic capabilities. Therefore, many members of DPANN may be obligately dependent on symbiotic interactions with other organisms and may even include novel parasites. In this contribution, we review the current knowledge of the gene repertoires and lifestyles of members of this group and discuss their placement in the tree of life, which is the basis for our understanding of the deep microbial roots and the role of symbiosis in the evolution of life on Earth.

## Teaser

We review current knowledge on the diversity and genomic potential of the only recently discovered enigmatic and potentially symbiotic DPANN archaea, discuss insights gained from functional studies of host–symbiont systems involving DPANN archaea as well as summarize controversies regarding the placement of the various DPANN lineages in the tree of life and thus the role of this putative radiation in the early evolution of life on Earth.

## INTRODUCTION

**Figure 1.**
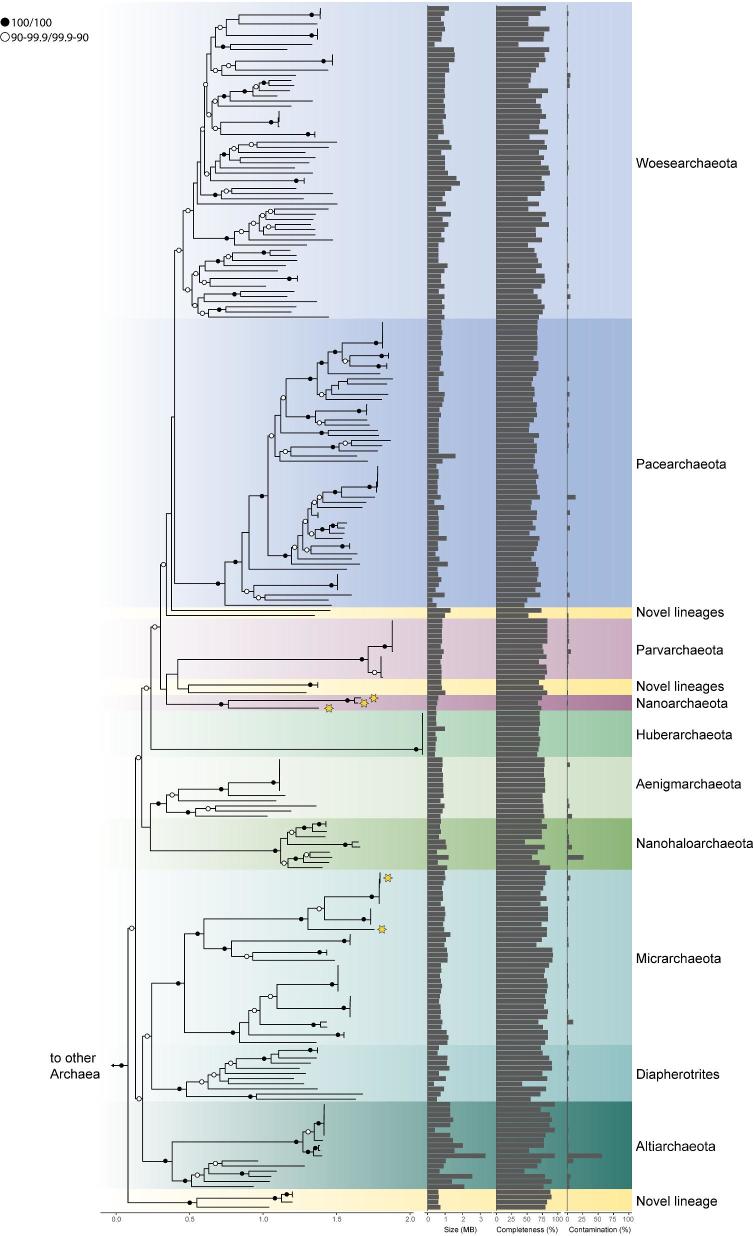
Phylogenetic tree displaying currently available taxonomic diversity of DPANN genomes. Tree was generated using the RP15 pipeline published previously in Zaremba-Niedzwiedzka *et al.* (2017) and is based on maximum-likelihood analyses in IQ-tree using the LG + C60 + F model of evolution. SH-values and bootstraps were calculated using a SH-like approximate likelihood ratio test (Guindon *et al.*^2010^) and ultrafast bootstrap support values (Minh *et al.*^2013^), respectively. Black and white circles indicate support values of 100/100 and 99.9–90/90–99.9, respectively. Scale bars indicate the average number of substitutions per site. The phylogeny represents bins available at NCBI before 23 March 2018 and indicates the genome size (for closed genomes), the bin completeness and contamination (for MAGs). The stars highlight genomes from DPANN members existing in co-cultures. Corresponding information for these genomic bins is available in Supplementary Table S1 (Supporting Information), the tree including taxa names is available as Supplementary Fig. S1 (Supporting Information) and the tree file is provided in Supplementary Data 1 (Supporting Information).

Antoni van Leeuwenhoek is often credited for the discovery of bacteria, which he visualized for the first time through a microscope of his own design in 1676 (Leeuwenhoek 1677). It took another 300 years before Carl Woese and George Fox inferred that methane-producing microorganisms (i.e. methanogens)—thought to be bacteria—in fact represent members of a separate domain of life, now referred to as Archaea (Woese and Fox 1977; Woese, Kandler and Wheelis 1990). Burgeoning methodologies for the sequencing of nucleic acids and the reconstruction of phylogenies showed that, in addition to methanogens, archaea included a range of extremophilic organisms: halophiles, acidophiles and hyperthermophiles. The assumption that most Archaea inhabit extreme environments of limited global significance prevailed until the early 1990s, which saw the first reports of archaeal organisms detected in marine waters (DeLong 1992; Fuhrman, McCallum and Davis 1992). Various lineages of archaea are now known to be globally distributed and prevalent in marine pelagic and benthic ecosystems as well as soils (DeLong, Pace and Kane 2001) and were shown to be of utmost importance for our understanding of the origin of eukaryotes (Spang *et al.*^2015^; Eme *et al.*^2017^; Zaremba-Niedzwiedzka *et al.*^2017^). Surprisingly, archaea were not known to include pathogens or endosymbionts and were for a long time thought to comprise predominantly free-living organisms. This view changed with the discovery of the ultrasmall ectosymbiotic archaeon *Nanoarchaeum equitans*, whose growth is obligately dependent on its archaeal host *Ignicoccus hospitalis* (Huber *et al.*^2002^). In agreement with its host dependency, *N. equitans* was found to have a small reduced genome encoding a limited set of metabolic functions and was suggested to be the first member of a separate archaeal phylum referred to as Nanoarchaeota (Huber *et al.*^2002^; Waters *et al.*^2003^). Since then, the application of single-cell and metagenomic approaches has helped to gradually refine our picture of archaeal phylogenetic diversity (Adam *et al.*^2017^; Spang, Caceres and Ettema 2017) and unveiled the genomes of a large amount of additional nanosized and/or genome-reduced archaeal lineages (Fig. 1, Supplementary Fig. S1, Supplementary File 1, Supporting Information) (Rinke *et al.*^2013^; Castelle *et al.*^2015^; Castelle and Banfield 2018; Probst *et al.*^2018^). In initial analyses, these genome-reduced archaea were suggested to form a monophyletic and deep-branching archaeal superphylum, which included Nanoarchaeota and was collectively referred to as DPANN—an acronym for the different phyla known at the time, the Diapherotrites, Parvarchaeota, Aenigmarchaeota, Nanoarchaeota and Nanohaloarchaeota (Rinke *et al.*^2013^). In contrast to *N. equitans*, which was enriched in a co-culture with its hosts and has been studied extensively (Huber *et al.*^2002^; Waters *et al.*^2003^; Jahn *et al.*^2004^, ^2008^; Burghardt *et al.*^2009^; Giannone *et al.*^2015^; Mohanty *et al.*^2015^; Heimerl *et al.*^2017^), most of our knowledge of the biology of other DPANN archaea is derived from genomic data (Castelle and Banfield 2018). Therefore, much has to be learned about these enigmatic archaea and the coming years will certainly witness a reappraisal of the extent of symbiotic interactions involving archaea.

In this contribution, we review the history of the discovery of DPANN archaea, our current understanding of their metabolic potential, features of their reduced genomes, their host–symbiont interactions as well as their phylogenetic diversity and evolutionary history. This will provide a foundation for our understanding of the symbiotic nature of these archaea and can guide prospective efforts to unveil both the functional importance of these extremely diverse but understudied organisms and their role in the early evolution and diversification of Archaea.

## THE DISCOVERY OF ARCHAEA WITH SMALL GENOMES AND CELL SIZES

The first member of the Nanoarchaeota, *N. equitans*, was discovered in an enrichment culture established from samples collected at a hydrothermal site north of Iceland (Huber *et al.*^2002^). This organism is characterized by small cells of just 400 nm in diameter and encodes one of the smallest known archaeal genomes (0.49 Mb) (Huber *et al.*^2002^; Waters *et al.*^2003^) (Fig. 1, Supplementary Table S1, Supplementary Fig. S1, Supplementary File 1, Supporting Information). A few years later, slightly larger genomes (0.64–1.08 Mb) of other ultrasmall archaea with cell volumes as low as 0.009 μm^3^ were recovered from an acid mine drainage and initially referred to as archaeal Richmond Mine acidophilic nanoorganisms (ARMAN), but later renamed to Parvarchaeota and Micrarchaeota (Comolli *et al.*^2009^; Baker *et al.*^2010^; Baker and Dick 2013). An additional lineage of nanosized archaea (0.1–0.8 μm) named Nanohaloarchaea was found in hypersaline environments (Ghai *et al.*^2011^; Narasingarao *et al.*^2012^; Rinke *et al.*^2013^; Vavourakis *et al.*^2016^) and initially thought to comprise a sister lineage of Haloarchaea (Narasingarao *et al.*^2012^). Subsequent analyses indicated alternative positions for this lineage and raised the possibility that Nanohaloarchaeota belong to DPANN (Rinke *et al.*^2013^); however, their phylogenetic placement in the Euryarchaeota is still debated (Aouad *et al.*^2018^) (see sections below). The use of single-cell genomics unveiled an even larger diversity of small archaea in brackish/freshwater and hydrothermal environments and led to the description of Diapherotrites and Aenigmarchaeota (Rinke *et al.*^2013^), the latter of which were originally known as DSEG archaea (Takai *et al.*^2001^). DHVE-5 and 6 archaeal groups, which were first described by 16S rRNA gene analyses (Takai and Horikoshi 1999; Durbin and Teske 2012), were renamed to Pacearchaeota and Woesearchaeota, upon the recovery of the first metagenome-assembled genomes (MAGs) from an aquifer (Castelle *et al.*^2015^). Pacearchaeota and Woesearchaeota seem to represent the most ubiquitously distributed lineages within the DPANN and have been detected in groundwater (Castelle *et al.*^2015^), freshwater lakes (Ortiz‐Alvarez and Casamayor 2016), ocean sediments (Durbin and Teske 2010) and hydrothermal vents (Takai and Horikoshi 1999). Recently, Woesearchaeota have even been identified in permafrost samples and the human microbiome (Shcherbakova *et al.*^2016^; Koskinen *et al.*^2017^). The most recent addition to the DPANN superphylum is the Huberarchaeota, for which MAGs have been recovered from a CO_2_-driven geyser (Probst *et al.*^2018^). While the Altiarchaeota have been first placed within the Euryarchaeota, several phylogenetic analyses indicate that they may affiliate with the DPANN archaea (Spang, Caceres and Ettema 2017; Castelle and Banfield 2018; Castelle *et al.*^2018^) (see sections below). Their first representative, *Cand*. Altiarchaeum hamiconexum (formerly known as SM1 Euryarchaeon), was discovered in a sulfidic spring (Probst *et al.*^2013^, ^2014^), where it forms ‘string-of-pearls’, a biofilm community where the archaeon inhabits the interior and a filamentous bacterial species dominates the exterior of the ‘pearls’ (Rudolph, Wanner and Huber 2001). However, not all Altiarchaeota appear to form such biofilms as suggested by the recovery of altiarchaeotal genomes from river sediments, springs and lakes (Bird *et al.*^2016^). Altogether, DPANN archaea represent an extremely diverse putative superphylum that comprises more than 10 phylum-level lineages most of which share small cell and genome sizes as common features (Fig. 1).

## WHAT IS ENCODED BY THE SMALL GENOMES OF DPANN ARCHAEA?

### The metabolic potential of DPANN archaea

While the metabolic potential of DPANN archaea appears to vary considerably both between and within DPANN lineages, most of these organisms are characterized by sparse metabolisms with limited catabolic and anabolic capabilities indicating that at least some members may represent obligate symbionts (Castelle and Banfield 2018; Castelle *et al.*^2018^). In this section, we will briefly summarize the main metabolic features predicted for DPANN archaea and refer the interested reader to excellent publications offering comprehensive overviews for more details (Castelle *et al.*^2015^, ^2018^; Castelle and Banfield 2018; Chen *et al.*^2018^; Liu *et al.*^2018^).

#### Energy metabolism

Most of the currently available DPANN genomes lack genes encoding known components of electron transport chains capable of generating a proton/sodium-motive force, which drives ATP production via a membrane-bound ATP synthase (Castelle and Banfield 2018; Castelle *et al.*^2018^). In contrast, genes encoding enzymes involved in the formation of fermentation products including lactate, formate, ethanol, acetate (Castelle and Banfield 2018) as well as butyrate (Chen *et al.*^2018^) are detected in various genomes of DPANN archaea, suggesting that many of the currently known members are anaerobes with the potential to use substrate-level phosphorylation as main mode of energy conservation. Representatives of the Altiarchaeota could constitute a notable exception (Probst *et al.*^2014^; Bird *et al.*^2016^). These org-anisms have been hypothesized to rely on a putative ferredoxin-dependent complex 1-like oxidoreductase to generate a proton-motive force and drive ATP synthesis via a membrane-bound ATP synthase. Although several DPANN archaea encode subunits of putative ATP synthases, these protein complexes might not always be functional as suggested by the structural and biophysical investigations of the A_3_B_3_ core complex of *N. equitans* (Mohanty *et al.*^2015^). Yet, some genomes assigned to Parvarchaeota and Micrarchaeota have been reported to encode putative components of an aerobic electron transport chain and a canonical A-type ATP synthase besides the fermentation pathways common to most DPANN, which indicate an ability for both aerobic and anaerobic metabolism (Baker *et al.*^2010^; Castelle and Banfield 2018; Castelle *et al.*^2018^; Chen *et al.*^2018^).

#### Catabolism

Some fermentation pathways predicted to operate in DPANN archaea are fed by pyruvate and others by acetyl-CoA. Homologs of enzymes converting pyruvate to acetyl-CoA (i.e. pyruvate dehydrogenase and pyruvate-ferredoxin oxidoreductase) appear to be broadly distributed across the DPANN superphylum (Castelle and Banfield 2018; Castelle *et al.*^2018^). Yet, components of this minimal energy metabolism (i.e. pyruvate-metabolizing enzymes) were absent in some DPANN genomes assigned to Woesearchaeota (Castelle *et al.*^2015^), Mamarchaeota (Castelle and Banfield 2018) and Nanoarchaeota (Podar *et al.*^2008^), raising questions about the potential source of energy in these organisms: e.g. is acetyl-CoA directly taken up by these organisms? In contrast, various organisms across the DPANN superphylum were predicted to operate additional catabolic pathways leading to the production of pyruvate and acetyl-CoA (Narasingarao *et al.*^2012^; Castelle *et al.*^2015^, ^2018^; Castelle and Banfield 2018; Chen *et al.*^2018^; Liu *et al.*^2018^). This includes the Embden–Meyerhof–Parnas pathway, an incomplete Entner–Doudoroff pathway, the beta-oxidation pathway and a RubisCO-dependent nucleoside degradation pathway (Sato, Atomi and Imanaka 2007; Aono *et al.*^2015^), suggesting that at least some DPANN archaea have the ability to conserve energy from the oxidation of hexoses, fatty acids and nucleosides. Further inferred catabolic capabilities include the depolymerization of oligosaccharides and polysaccharides in Nanohaloarchaeota, Micrarchaeota, Pacearchaeota, Parvarchaeota and Woesearchaeota (Narasingarao *et al.*^2012^; Castelle *et al.*^2015^; Chen *et al.*^2018^); the depolymerization of proteins in Diapherotrites, Micrarchaeota, Parvarchaeota and Woesearchaeota (Castelle *et al.*^2015^; Youssef *et al.*^2015^; Chen *et al.*^2018^; Liu *et al.*^2018^); the utilization of glycerol in Parvarchaeota (Chen *et al.*^2018^); the degradation of polyhydroxybutyrate in Diapherotrites (Youssef *et al.*^2015^); and, tentatively, the oxidation of ferrous iron in Parvarchaeota (Chen *et al.*^2018^). Catabolic pathways inferred to be encoded by altiarchaeotal genomes generate CO_2_ instead of organic acids. The former is suggested to derive from the oxidation of C1 carbon compounds, i.e. carbon monoxide and formate (Probst *et al.*^2014^; Bird *et al.*^2016^).

#### Anabolism

Many genomes of the DPANN archaea are characterized by the absence of genes encoding the enzymes of canonical, primary biosynthetic pathways for amino acids, purines, pyrimidines, lipids and vitamins (Castelle and Banfield 2018; Castelle *et al.*^2018^). Furthermore, many genomes lack genes encoding known components of the tricarboxylic acid (TCA) cycle and both oxidative and non-oxidative variants of the pentose phosphate pathway. In spite of these reduced biosynthetic capabilities, some members of the DPANN superphylum may synthesize some macromolecule building blocks (e.g. purine and pyrimidine biosynthetic pathways may operate in some Diapherotrites, Micrarchaeota, Aenigmarchaeota, Nanoarchaeota and Woesearchaeota), and some genomes assigned to Micrarchaeota and Parvarchaeota were recently reported to encode near-complete TCA cycles (Baker *et al.*^2010^; Krause *et al.*^2017^; Chen *et al.*^2018^). Contrasting with the apparent dependence of most DPANN organisms on reduced carbon compounds, representatives of the Altiarchaeota are autotrophs using a modified Wood–Ljungdahl pathway to fix carbon dioxide (Probst *et al.*^2014^).

**Figure 2.**
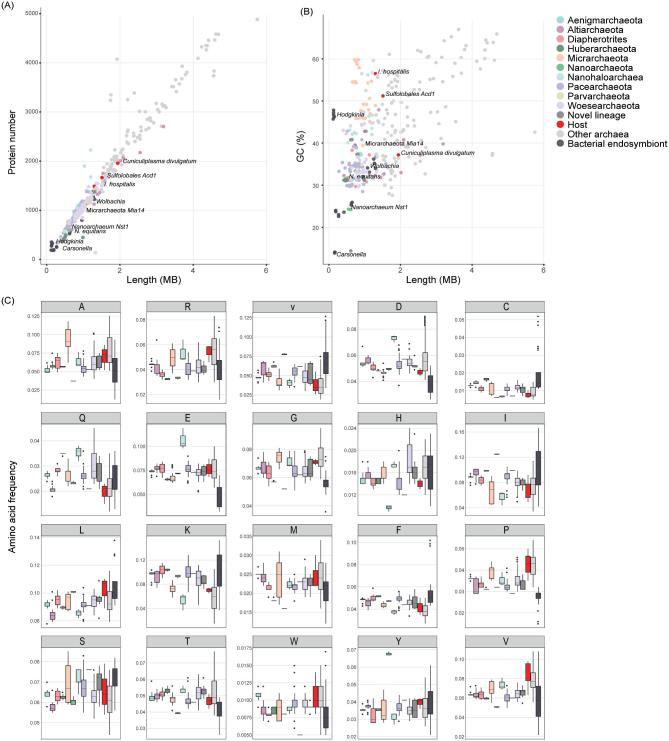
General genome characteristics of DPANN archaea. Dot plots displaying total protein numbers (A) and GC content (B) versus genome size for DPANN genomes as compared to other archaeal representatives as well as selected endosymbiotic bacteria (see also Supplementary Table S1, Supporting Information). (C) Average amino acid frequencies of members of the DPANN archaea compared to their hosts, other archaea and bacterial endosymbionts (see also Supplementary Table S2, Supporting Information). Frequencies are shown for each individual amino acid, which are listed in their single letter code. Boxplot shows the median, the first and third quartiles, the upper/lower whiskers that extend from the hinge to the largest/smallest value no further than 1.5× of the interquartile range from the hinge as well as outliers, which are represented as dots. Boxplots were generated with R v3.5.0 using the package ggplot2.

The general paucity of genes encoding homologs of known metabolic enzymes in at least some genomes of DPANN organisms is remarkable considering that comprehensive inventories of metabolic pathways can be reconstructed from genomes of bacterial symbionts, such as *Mycoplasma pneumoniae* (Yus *et al.*^2009^; Wodke *et al.*^2013^) or *M. genitalium* (Karr *et al.*^2012^), which possess, like DPANN archaea (Fig. 2A), few protein-coding genes. Current observations suggest that many members of the DPANN archaea must acquire multiple essential metabolites externally, consistent with the experimentally validated host-associated lifestyle of some DPANN organisms (see below) (Huber *et al.*^2002^; Jahn *et al.*^2008^; Golyshina *et al.*^2017^; Krause *et al.*^2017^). Nevertheless, the extent of the dependency of DPANN on the provision of essential metabolites remains unclear since many of the proteins encoded in their genomes have no known function, raising the question of whether DPANN organisms encode novel enzymes driving canonical or entirely new metabolic pathways.

### Other genomic features of DPANN archaea

In spite of these reduced metabolic capabilities and in contrast to findings in some bacterial endosymbionts (Moran and Bennett 2014), reductive genome evolution in DPANN archaea does not seem to coincide with the loss of the informational processing genes as most members of this group encode core informational processing machineries (Castelle and Banfield 2018). However, the occurrence of split genes encoding some proteins involved in informational processing, such as reverse gyrase and tRNA synthetases described in Nanoarchaeota and Micrarchaeota (Waters *et al.*^2003^; Randau, Pearson and Söll 2005; Randau *et al.*^2005^, Baker *et al.*^2010^), or the diphthamide biosynthesis protein Dph1/2 in some members of the Parvarchaeota, Pacearchaeaota, Woesearchaeota and Altiarchaeota (Narrowe *et al.*^2018^), may represent signs of reductive genome evolution in some of these lineages. Initially, split genes in Nanoarchaeota were proposed to represent an ancestral feature (Di Giulio 2006), thought to coincide with an early divergence of Nanoarchaeota (Waters *et al.*^2003^; Podar *et al.*^2013^). However, more recent analyses of additional members of Nanoarchaeota have led to the suggestion that split genes originated later in only some representatives of Nanoarchaeota with particularly reduced genomes (Podar *et al.*^2013^). This view is supported by the observation that some of the proteins encoded by split genes in *N. equitans*, such as DNA polymerase I, topoisomerase I and alanyl-tRNA synthetase, are encoded by a single gene in the close relative *Nanoarchaeota* Nst1 (Podar *et al.*^2013^) and do not reveal a deep-branching position in phylogenetic analyses (Andersson, Sarchfield and Roger 2005; Furukawa *et al.*^2017^). On the other hand, the primase found in various DPANN including *N. equitans*, *Parvarchaeum* ARMAN-5 and *Cand*. Nanosalinarum sp. (Raymann *et al.*^2014^) consists of only one subunit encoded by a single gene, while this enzyme consists of a catatalytic subunit (PriS) and an accessory subunit (PriL) encoded by two distinct genes in other archaea. While *Micrarchaeota* ARMAN-2 has a canonical archaeal primase encoded by two genes, the atypical primase of most DPANN archaea may represent a putative synapomorphy (a shared derived character) for a subset of DPANN lineages. However, subsequent phylogenetic analyses that include a wider representation of primases encoded by the various recently described DPANN lineages will be necessary to resolve the evolutionary history of this protein family and thereby shed more light onto replication machineries of DPANN archaea.

## HOST–SYMBIONT SYSTEMS INVOLVING DPANN ARCHAEA

The limited metabolic capacities of most DPANN archaea described above suggest that various of these organisms have a predominately symbiotic lifestyle. However, to date only few stable co-cultures of DPANN archaea with their hosts have been obtained: *N. equitans* with *I. hospitalis* (Huber *et al.*^2002^; Waters *et al.*^2003^), *Nanoarchaeota* Nst1 (later renamed to *Nanobsidianus stetteri*) with *Sulfolobales* Acd1 (Podar *et al.*^2013^; Munson-McGee *et al.*^2015^), *Cand.* Nanopusillus acidilobi with *Acidilobus* sp. 7A (Wurch *et al.*^2016^), *Cand.* Micrarchaeota Mia14 with *Cuniculiplasma divulgatum* PM4 (Golyshina *et al.*^2017^), *Micrarchaeota* (ARMAN-1) A_DKE with *Cuniculiplasma* sp*.* C_DKE (Krause *et al.*^2017^) and *Cand.* Nanoclepta minutus Ncl-1 with *Zestosphaera tikiterensis* NZ3T (St. John *et al.*^2018^). Furthermore, a potential interaction of *Cand.* Huberarchaeum crystalense with members of the Altiarchaeota was suggested based on co-varying cell abundance profiles and microscopic imaging (Probst *et al.*^2018^). A recent meta-analysis of publicly available archaeal 16S rRNA gene sequences revealed the potential co-occurrence of operational taxonomic units (OTUs) derived from Woesearchaeaota with those of Methanomicrobia and Methanobacteria and may indicate interactions between members of these groups (Liu *et al.*^2018^). Interestingly, some protein sequences of DPANN archaea, such as aminoacyl tRNA synthetases of Nanoarchaeota, Parvarchaeota, Woesearchaeota and Micrarchaeota and the diphthamide biosynthesis protein Dph5 of Woesearchaeota, cluster with eukaryotic homologs in phylogenetic analyses (Andersson, Sarchfield and Roger 2005; Furukawa *et al.*^2017^; Narrowe *et al.*^2018^). It is tempting to speculate that this could imply a symbiotic relationship of some DPANN archaea with eukaryotes, considering that horizontal gene transfer seems to be common between DPANN symbionts and their hosts, such as between *N. equitans* and *I. hospitalis* (Podar *et al.*^2008^) or *Cand.* Micrarchaeota Mia14 and *C. divulgatum* PM4 (Golyshina *et al.*^2017^). In light of these findings, it is interesting to note that a recent study detected potential Nanoarchaeota-related organisms in an enrichment culture consisting of a few bacterial species as well as the protist *Carpediemonas frisia* (Hamann *et al.*^2017^). Finally, 16S rRNA gene sequences assigned to Pacearchaeota and Woesearchaeota were found to positively correlate with bacterial communities (Ortiz‐Alvarez and Casamayor 2016). These findings might suggest that some DPANN archaea interact with either bacterial or eukaryotic partners. However, further analyses are necessary to confirm these observations and determine whether they indicate metabolic interactions or physical associations between specific bacteria or eukaryotes with DPANN. Intriguingly, current analyses suggest that at least some DPANN archaea, for example members of Nanohaloarchaeota and *Cand*. Iainarchaeum andersonii (phylum Diapherotrites) (Narasingarao *et al.*^2012^; Youssef *et al.*^2015^) as well as Altiarchaeota (Bird *et al.*^2016^), may be capable of leading an independent lifestyle. Altogether, this highlights the multitude of lifestyles found across DPANN archaea and suggests that adaptations to symbiotic growth modes may have evolved several times independently.

The metabolic dependencies of DPANN archaea on their hosts have been studied extensively using *N. equitans* with *I. hospitalis* as a model system. *N equitans* displays a high host specificity and can only be grown in co-culture with *I. hospitalis*. Furthermore, while *N. equitans* can be separated from its host, its cells appear unable to proliferate (Huber *et al.*^2002^; Jahn *et al.*^2008^). These findings suggest a strong host dependency and the existence of a specific, yet unknown, recognition system. It is still debated whether this interaction is of a mutualistic or parasitic nature as there have not been any experimental setups that study this interaction under natural conditions. In agreement with the limited gene repertoire encoding for core metabolic pathways of *N. equitans* (Waters *et al.*^2003^), this organism seems to obtain various metabolites from *I. hospitalis* rather than from the environment (Jahn *et al.*^2004^, ^2008^; Hamerly *et al.*^2015^). Both organisms share essentially the same lipid composition (Jahn *et al.*^2004^) and amino acid labeling studies were unable to distinguish their labeling patterns (Jahn *et al.*^2008^), suggesting that *N. equitans* obtains its membrane lipids and amino acids from its host. Furthermore, the likely inactive ATPase of *N. equitans* raises the question of how it obtains ATP (Lewalter and Müller 2006; Mohanty *et al.*^2015^). The unique membrane system of *I. hospitalis*, consisting of an inner- and outer membrane separated by a large periplasmic space and being one of the few examples of an energy-conserving outer membrane, is debated to play an essential role in energy conservation of *N. equitans* (Küper *et al.*^2010^). Specifically, the presence of an ATPase and H_2_:sulfur oxidoreductase in the outer membrane of *I. hospitalis* suggests that ATP is generated in the periplasm of this organism and might be accessible to *N. equitans* (Küper *et al.*^2010^; Mayer *et al.*^2012^). Around 10% of the proteome changes upon interaction of *I. hospitalis* with *N. equitans* including an upregulation of proteins related to energy conservation, cell cycle control and membrane modification (Giannone *et al.*^2011^, ^2015^). More specifically, the upregulation of an ATP synthase, NiFe-hydrogenase or pyruvate:ferredoxin oxidoreductase might reflect the higher energy demands imposed on the host. While *N. equitans* cells do not appear to contain significant amounts of host proteins (Giannone *et al.*^2011^), it was shown that the overall metabolite pool recovered from the co-culture is less concentrated than in the host alone, implicating that *N. equitans* stimulates the consumption of *I. hospitalis* metabolites (Hamerly *et al.*^2015^).

**Figure 3.**
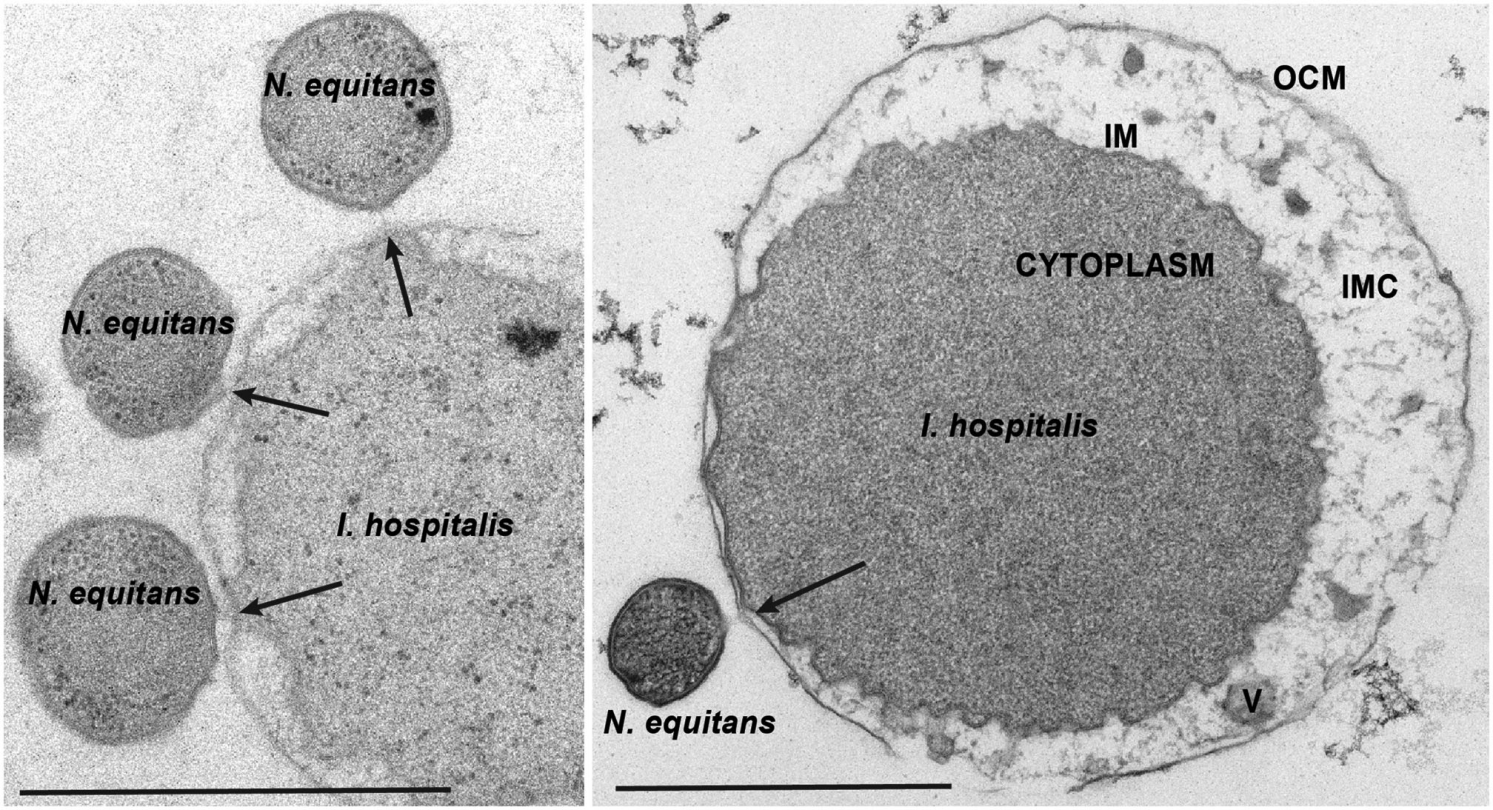
Transmission electron micrographs (TEM) of *I. hospitalis* and *N. equitans* interaction*.* This TEM of ultrathin sections of *I. hospitalis* and *N. equitans* display the interactions between this host–symbiont system and are based on a slightly modified and updated version of Fig. 4 originally published in Jahn *et al.* (2008) and kindly provided by Harald Huber. OCM: outer cellular membrane, IMC: intermembrane compartment, IM: inner membrane. Bars: 1 μm.

Even though studies of the interactions between *I. hospitalis* and *N. equitans* have increased our understanding of the associations involving DPANN archaea, it is still unclear how metabolites are interchanged. In the case of *N. equitans*, two modes of interaction with *I. hospitalis* are proposed: (a) direct periplasmic contact and (b) indirect contact between cells via thin fibers (Junglas *et al.*^2008^) (Fig. 3). The isolation of the contact side has identified hypothetical subunits of the Sec protein translocase complex (SecD), the A1A0-ATPase as well as potential transporters of *I. hospitalis* and *N. equitans.* These components might mediate the transfer of metabolites (Burghardt *et al.*^2009^) or enzymes, such as the fatty acid-CoA ligase encoded by *I. hospitalis*, into the cytoplasmic space of *N. equitans* (Heimerl *et al.*^2017^). Intriguingly, hosts of DPANN archaea can differ dramatically in their membrane architecture, as is the case for *I. hospitalis* and *Sulfolobales* Acd1, the hosts of *N. equitans* and *Nanoarchaeota* Nst1, respectively. While *I. hospitalis* has a double membrane, Acd1 likely encodes an S-layer (Podar *et al.*^2013^), indicating that even closely related DPANN archaea might have evolved different means to interact with their respective hosts. In line with these observations, a multitude of cell surface structures have been observed in different DPANN lineages. For example, ARMAN-like cells (likely belonging to both Micrarchaeota and Parvarchaeota) might establish direct cytoplasmic contacts via synapse-like or tubular structures or utilize needle-like penetration mechanisms (Comolli *et al.*^2009^; Baker *et al.*^2010^; Comolli and Banfield 2014). Furthermore, hami (‘grappling hook’-like structures) of Altiarchaeota were suggested to be employed for the attachment to other cells (Probst and Moissl-Eichinger 2015). It is however unknown whether these structures also play a role in the interaction between members of the Altiarchaeota and *Cand*. Huberarchaeum crystalense (Probst *et al.*^2018^).

Altogether, studying the symbiotic lifestyle of DPANN archaea has provided us with a better understanding of their metabolic dependencies, multitude of potential hosts and means of interactions. Nevertheless, and also considering the large diversity of DPANN-affiliated lineages, much has to be learned about the interactions these organisms are engaged in. Studying the variety of symbiotic lifestyles found across DPANN archaea using both genomics and microbiological approaches will provide additional insights into the diversity of mechanisms characterizing archaeal symbioses.

## THE EVOLUTION OF DPANN ARCHAEA AND THEIR POSITION IN THE TREE OF LIFE

Ever since the discovery of *N. equitans*, the phylogenetic placement of DPANN lineages in the archaeal tree has been uncertain and is the subject of controversies. Initial phylogenies placed *N. equitans* as an outgroup to the Euryarchaeota and Crenarchaeota, the two main lineages of archaea known at the time (Waters *et al.*^2003^). Subsequent work found some tentative support for a relationship of *N. equitans* with Thermococcales (Brochier *et al.*^2005^), although this placement was not recovered in later analyses (Brochier-Armanet *et al.*^2008^). Improved taxonomic sampling usually helps to resolve the phylogenetic placement of unstable taxa (Graybeal and Cannatella 1998), and the discovery of a broad diversity of additional DPANN lineages (Rinke *et al.*^2013^; Castelle *et al.*^2015^) provided an injection of much-needed genome data to the debate. Various phylogenies of this expanded genome sampling suggested that DPANN archaea form a monophyletic group at the base of the archaeal tree (Rinke *et al.*^2013^; Spang *et al.*^2013^; Castelle *et al.*^2015^; Saw *et al.*^2015^; Spang, Caceres and Ettema 2017; Williams *et al.*^2017^), although alternative topologies have been observed (Petitjean *et al.*^2015^). Thus, none of these analyses has definitively resolved the phylogenetic placement of the different DPANN lineages, hampering our understanding of the metabolic gene repertoire of the last common ancestor of the Archaea as well of the role of symbioses in the evolution and diversification of the Archaea.

### The challenge of determining the phylogenetic placement of symbionts

**Figure 4.**
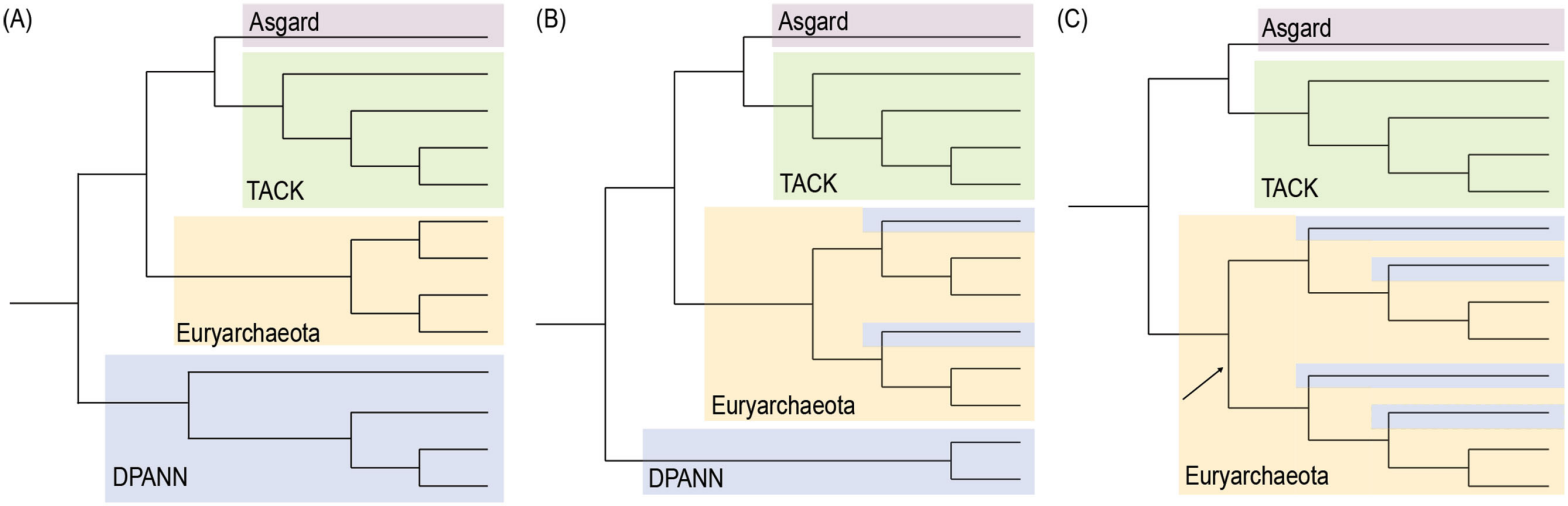
Illustration of controversially discussed placements of DPANN lineages in the archaeal part of the tree of life. While most studies indicate that DPANN represent a deep-branching archaeal superphylum (A), few studies have suggested that some (B) or all DPANN lineages (C) could represent fast-evolving archaeal taxa, which are artificially attracted to the base of the archaeal tree while in fact affiliating with other archaeal lineages.

Based on published analyses, at least three scenarios for DPANN phylogeny seem possible (Fig. 4). First, DPANN might represent a monophyletic clade branching at the base of the Archaea; this position is consistent with most of the published trees that include representatives from all known DPANN groups (Rinke *et al.*^2013^; Spang *et al.*^2013^; Castelle *et al.*^2015^; Saw *et al.*^2015^; Spang, Caceres and Ettema 2017; Williams *et al.*^2017^). Secondly, some DPANN lineages may form a clade at the base of Archaea, while others would be secondarily derived lineages that group erroneously with the basal clade as a result of phylogenetic artifacts (Aouad *et al.*^2018^). Finally, all of the DPANN lineages might be secondarily derived from within other archaeal groups, with their apparent monophyly being the result of phylogenetic artifacts such as long branch attraction (LBA). In LBA, long branches are erroneously grouped together in a tree even though they are not closely related (Bergsten 2005). The causes of LBA and the conditions under which it occurs are poorly understood, but the basic problem is that evolution along long branches is difficult to model. When multiple changes occur at a single amino acid site, it can be difficult to determine whether identical states represent evidence of recent common ancestry—that is, synapomorphies—or are the result of convergent evolution. In particular, parasites and symbionts are thought to experience different selective pressures compared to free-living organisms (Moran and Bennett 2014). For example, the switch to a host-associated lifestyle in bacteria often coincides with gene loss, genomic and cellular reduction, an elevated rate of sequence evolution and mutation-driven drift towards very low or very high GC contents (Clark, Moran and Baumann 1999; Moran 2003; Toft and Andersson 2010; Moran and Bennett 2014). In general, these characteristics can cause phylogenetic artifacts that can impede the exact phylogenetic placement of symbionts in the tree of life if not modeled carefully (Moran 1996). In the case of DPANN, the suspicion is that a large number of convergent amino acid changes associated with the transition from a free-living to host-associated lifestyle might cause these organisms to artifactually group together in a deep-branching clade. If so, it has been argued (Aouad *et al.*^2018^) that increased sampling of DPANN might worsen, rather than ameliorate, the problems of LBA.

While GC content and amino acid frequencies of DPANN archaea do not appear to be as strongly biased as those of bacterial endosymbionts, they differ to some extent from the average of other archaea (Fig. 2B and C, Supplementary Table S2, Supporting Information). It remains to be determined whether these differences lead to compositional effects that could cause systematic phylogenetic artifacts. In addition, the long branches of DPANN representatives in published phylogenetic trees may indicate that genomes of members of this group evolve faster than non-symbiotic archaea. Genomic features of DPANN archaea that could potentially contribute to accelerated evolutionary rates include diversity-generating elements (DGRs), CRISPR systems and transposons. In particular, DGRs allow to create massive amounts of sequence variation in selected ligand-binding sites of target proteins (Doulatov *et al.*^2004^; McMahon *et al.*^2005^) and seem to be prevalent in DPANN lineages such as Nanoarchaeota (Paul *et al.*^2015^), Pacearchaeota and Woesearchaeota (Handa *et al.*^2016^; Paul *et al.*^2017^) where they might play a role in cell–cell attachment and thus might be beneficial for dynamic host responses (Paul *et al.*^2017^). Furthermore, at least some members of the DPANN encode CRISPR-Cas9 systems (Burstein *et al.*^2017^), which coincides with the observation that some representatives of this group are subjected to viral infections (Comolli *et al.*^2009^; Martínez-García *et al.*^2014^). Frequent viral infections may influence genome dynamics due to an evolutionary arms race between viruses and hosts (Stern and Sorek 2011) and could thereby contribute to increased rates of evolution. Finally, the recent discovery of a duplicated transposon and localized single nucleotide polymorphisms in *Cand*. Forterra multitransposorum (Diapherotrites) suggests that homologous recombination could also affect genome evolution in DPANN archaea (Probst and Banfield 2018). However, these characteristics are not universally conserved across the DPANN superphylum. Additionally, the observed loss of genes coding for proteins involved in DNA repair machineries in bacterial endosymbionts (Moran, McCutcheon and Nakabachi 2008) does not seem to represent a defining feature of DPANN archaea (Castelle *et al.*^2018^), suggesting that means to control mutation rates might be retained in at least some members. Furthermore, several lineages that affiliate with DPANN in phylogenies, such as the Altiarchaeota, do not seem to represent obligate symbionts or are characterized by reductive genome evolution (Fig. 1, Supplementary Fig. S1, Supplementary File 1, Supporting Information). Therefore, it remains to be determined whether genome evolution of DPANN lineages is indeed characterized by fundamentally different rates as compared to other archaeal lineages and whether the potential host-associated lifestyle of members of this diverse clade contributes to the observed topology or whether DPANN monophyly represents a genuine signal.

### Current insights from phylogenetic analyses on the monophyly of DPANN archaea

To evaluate potential artifacts regarding the placement of DPANN in the tree of Archaea carefully, a number of authors have investigated the robustness of the DPANN clade using analyses that attempt to mitigate LBA. The CAT + GTR substitution model has been shown to be less susceptible to LBA artifacts (Lartillot, Brinkmann and Philippe 2007) because the probabilities of change among amino acid states are calculated per site, rather than averaged over the entire sequence alignment as in standard phylogenetic models (Lartillot and Philippe 2004). The monophyly of DPANN received maximal support, both when CAT + GTR was used to analyze raw amino acid sequences and when the alignment was recoded into four Dayhoff categories of biochemically similar amino acids (Williams *et al.*^2017^). Although the latter reduces information, this type of data recoding is a useful exploratory tool as it reduces substitutional saturation and compositional variation among the sequences and thereby allows a more accurate modeling of the data (Hrdy *et al.*^2004^; Susko and Roger 2007). The placement of the different DPANN clades relative to each other was also assessed by comparing DPANN phylogenies, obtained by excluding or including representatives of other archaeal lineages, respectively (Williams *et al.*^2017^). If DPANN monophyly is an LBA artifact, one may expect that the relationships within the group should be essentially random, and there is no reason to expect the same tree structure in the DPANN-only analysis. Yet, the results of both analyses were very similar and seem thus consistent with DPANN monophyly. However, it cannot be excluded that shared compositional biases between particular DPANN lineages could have caused the similar topologies of DPANN lineages relative to each other (Fig. 2B and C).

To date, the strongest evidence against DPANN monophyly comes from analyses that attempt to place DPANN taxa or sublineages into the tree of Archaea one at a time (Williams *et al.*^2017^; Aouad *et al.*^2018^). The assumption of this approach is that, if DPANN are indeed monophyletic, each individual member of the group should connect to a backbone phylogeny of other archaea in the same position when analyzed in isolation. If DPANN monophyly is instead an LBA artifact, individual taxa might be easier to place than the group as a whole, and different placements of the individual lineages might suggest that DPANN monophyly is artifactual. Williams *et al.* (2017) found that the Diapherotrites, Aenigmarchaeota and Woesearchaeota lineages branched basally when analyzed individually, but Nanoarchaeota, Nanohaloarchaeota and Pacearchaeota instead grouped within Euryarchaeota. Consistent with these results, Aouad *et al.* (2018) reported that Nanohaloarchaeota branched from the euryarchaeotal stem when the entire concatenated alignment was analyzed, but grouped with the Methanocellales (that is, within the Euryarchaeota) when including only slow-evolving sites. Finally, in this context it may also be worth considering the phylogenetic placement of Altiarchaeota. When analyzed individually, Altiarchaeota group within the Euryarchaeota (Probst *et al.*^2014^; Adam *et al.*^2017^), but this clade groups with DPANN when these are included in the analysis (Bird *et al.*^2016^; Hug *et al.*^2016^; Spang, Caceres and Ettema 2017). Taken together, these results demonstrate that the phylogenetic resolution of DPANN lineages is sensitive both to taxon sampling and to the methods of analysis used, and further work is needed to robustly place the DPANN archaea in the tree of life.

Previous difficult phylogenetic problems were ultimately solved through improved taxon sampling and the use of better-fitting phylogenetic models (Embley and Martin 2006), which account for both site-specific biochemical constraints and across-branch compositional heterogeneity. Thus, such approaches may also be of help to further assess the placement of DPANN lineages. While site- and branch-heterogeneous models have been described (Blanquart and Lartillot 2008; Heaps *et al.*^2014^; Williams *et al.*^2015^; Cherlin *et al.*^2018^) and implemented, they are currently computationally intractable even on modestly sized phylogenomic datasets. Therefore, speed-ups in the implementations of these methods, and the development of new, more efficient branch-and-site models, will be essential to help resolve DPANN phylogeny. Furthermore, the inclusion of a taxonomically broader dataset of potential DPANN lineages (Fig. 1) in careful phylogenetic analyses will help towards resolving the evolution of this enigmatic group of archaea. In particular, the assessment of the placement of Altiarchaeota, which do not show substantial indications for genome reduction, will be essential for anchoring the more derived DPANNs in the tree and for determining the degree to which convergent processes affect reductive genome evolution in Archaea.

## CONCLUSION AND OUTLOOK

The astounding diversity of available genomes of DPANN archaea (Fig. 1) and the currently limited set of characterized host–symbiont systems emphasize how much we still need to learn about the basic biology of these organisms. For example, important questions that need to be addressed in the coming years will be to determine which lineages are host-dependent and which are free-living; what are their host organisms and/or interaction partners and how much variation in terms of lifestyle, metabolism and gene content exists among these lineages. Certainly, the development and application of new methods will be essential to recover novel host–symbiont systems involving DPANN archaea (Jarett *et al.*^2018^) and start answering some of these questions. Insights gained will shed light on what appears to be—at the very least—a major way of living among archaea and provide a better basis for assessing the functional importance and ecological role of members of the DPANN. Clearly, the vast diversity of DPANN archaea, most of which may depend on interacting partners and the presence of members of this group in environments across the entire biosphere (Castelle and Banfield 2018), indicate that they may represent a non-negligible component of microbial food webs (Probst *et al.*^2018^).

Currently, we still face major difficulties to unequivocally determine the evolutionary origins of DPANN and thus the evolution of symbiosis in the archaeal domain of life. Taken at face value, recent phylogenies (Hug *et al.*^2016^; Castelle and Banfield 2018; Castelle *et al.*^2018^) suggest that the deepest split within both the archaeal and bacterial domains appears to be between a clade of relatively small, apparently genome-reduced lineages with various shared genetic features—i.e. the DPANN archaea on one side and the bacterial candidate phyla radiation (CPR) on the other side. This remains true, even if the taxonomic level of these lineages still represents a matter of debate (Parks *et al.*^2018^). At least in the case of the bacterial tree, it is important to note that the many previously described highly reduced, fast-evolving symbiotic and parasitic bacterial lineages do not group with CPR, suggesting that current phylogenetic methods do not invariably succumb to LBA when parasites and symbionts are included in the analysis. Clearly, determining whether the monophyletic clustering and deep placement of genome reduced organisms in the tree of life represent a genuine phylogenetic signal is one of the most challenging and important current questions in microbial evolution. Tackling these questions will allow us to illuminate and potentially considerably change our current understanding of the nature of the last common ancestor of Archaea and Bacteria as well as of the role of symbiosis in the evolution of life on Earth.

## Supplementary Material

Supplement Files
