# Supplementary figures and images for: Genomic diversity, lifestyles and evolutionary origins of DPANN archaea
Source: FEMS Microbiol Lett. 2019 Jan 9;366(2):fnz008. doi: 10.1093/femsle/fnz008 (PMC6349945; doi:10.1093/femsle/fnz008)

Figure S1

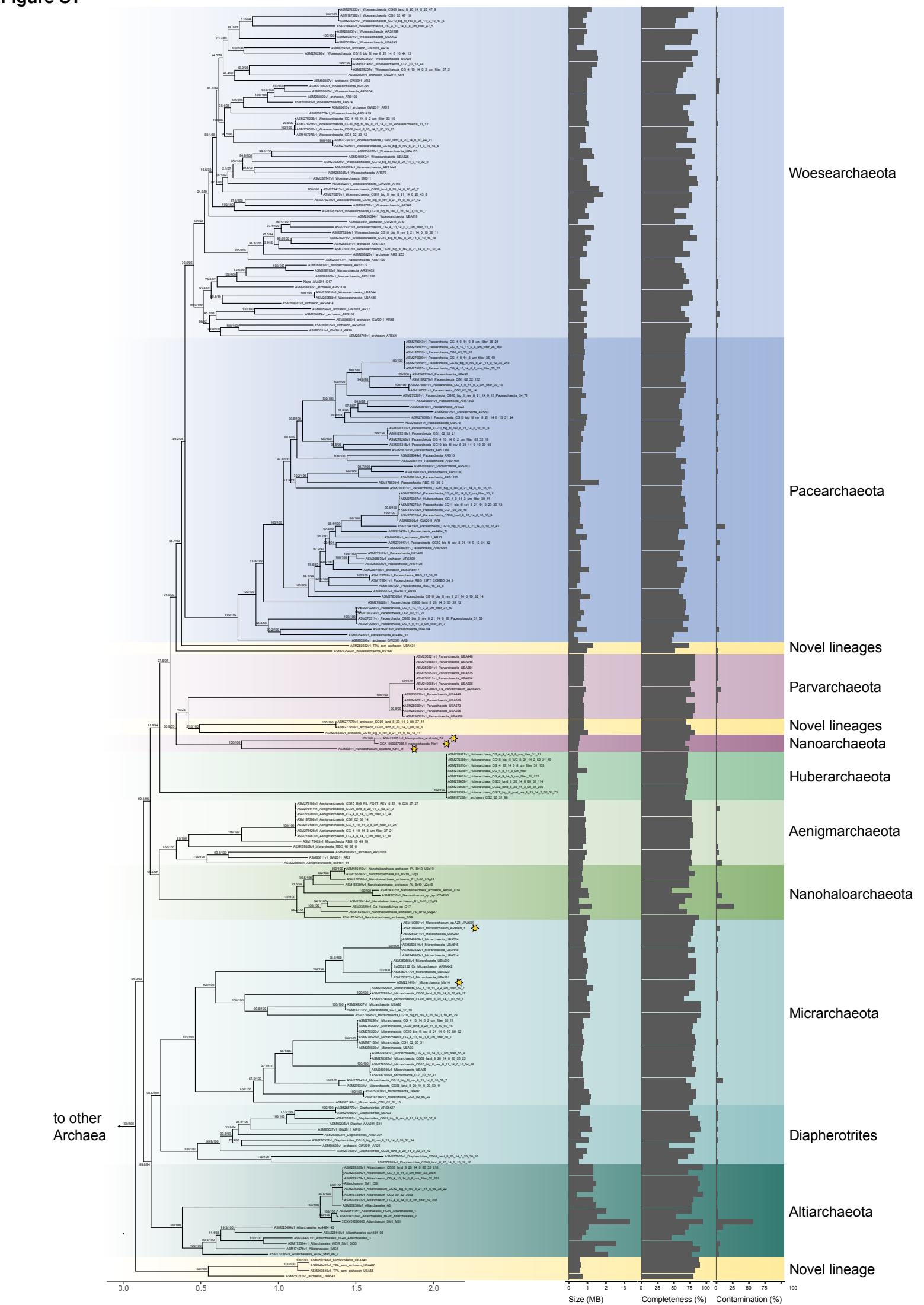

Supplement: Supplement Files [file fnz008_supplement_files.zip › Supplementary_Figure_1_R2.pdf]
